# Trace Element Concentrations in Lichens Collected in the Beskidy Mountains, the Outer Western Carpathians

**DOI:** 10.1007/s00128-015-1478-8

**Published:** 2015-01-30

**Authors:** Beata Klimek, Agata Tarasek, Joanna Hajduk

**Affiliations:** Faculty of Biology and Earth Sciences, Institute of Environmental Sciences, Jagiellonian University, Gronostajowa 7, 30-387 Kraków, Poland

**Keywords:** Air pollution, Atmospheric deposition, Bioindicators, Environmental monitoring, Heavy metals

## Abstract

The aim of the study was to assess trace metal air pollution in the Beskidy Mountains, the Outer Western Carpathians, Poland, with a widely used bioaccumulating organism, a lichen, *Hypogymnia physodes*. Lichens were collected at five stands (mountains) in parallel transect and analyzed for cadmium (Cd), copper (Cu), nickel (Ni), lead (Pb) and zinc (Zn) content. Concentrations of Cd, Pb and Zn in lichens were elevated, indicating moderate air pollution. The studied sites grouped in two clusters, with the three more contaminated sites being at the west end of the transect, and the two less polluted sites being situated more eastward. Such a pattern can be explained by the location of industrial centers and prevailing wind direction in southern Poland. The strongest correlation was noticed between Zn and Pb, which are known to occur jointly in ore deposits and are being processed in nearby Polish and Czech industrial regions.

The Carpathians are the second biggest mountain range in Europe after the Alps, harboring some of the most exceptional ecosystems of southern Poland. This mountain range is also a unique reservoir of many endemic plant and animal species (Grodzińska et al. 2004). Its outstanding biodiversity is protected by many national parks established in the region as well as other forms of nature protection, such as Natura 2000 (Olko et al. 2011). Increasing public concern about environmental protection issues has resulted in raised interest in human impacts on Carpathian forests, meadows and wetlands (Munteanu et al. 2014).

The aim of this study was to assess air pollution with trace metals in the Polish part of the Carpathians, i.e. in the Beskidy Mountains. Industrial heavy metal pollution has become a serious environmental problem all over the world. Even though they are typically considered a local issue, heavy metals when adsorbed to the surface of particulate matter (PM), can be transported in the atmosphere for long distances (Poličnik et al. 2004). In contrast to the common opinion that the Beskidy Mountains are relatively unpolluted, some studies have shown that they might be severely contaminated with heavy metals (Grodzińska et al. 1990). Since the 1990’s, numerous international environmental monitoring programs were introduced in the Carpathians (Bytnerowicz et al. 2003). Starting from 2003, a governmental nation-wide environmental monitoring programs have been introduced, supervised by the Chief Inspectorate of Environmental Protection, began collecting valuable data about air pollution, that subsequently was included in reports of the European Environmental Agency (EEA 2012). According to the data gathered to date, air pollution levels in Poland continue to decrease (see EEA Report No. 04/2012). Stations of monitoring system are located in city centers rather than in rural areas therefore data on pollution loads in less densely populated regions are scarce. In Poland there are four monitoring stations operating within the framework of the European Monitoring and Evaluation Program (EMEP) under the Convention on Long-range Transboundary Air Pollution. These stations, located in relatively remote and pollution-free regions, provide information about background concentrations of pollutants.

The EMEP station for measurements of background air pollution in mountain regions is located in southwest Poland, in another mountain range west of the Beskidy, namely the Sudetes. Due to the distance, the information collected by this station is not very representative for the Polish part of the Carpathians, meaning there is no recent data on trace metal air pollution in the Beskidy Mountains. The present study is an attempt to partially fill this gap.

Mountain massifs may block tropospheric air movements, which leads to high precipitation in these areas. Thus, mountain regions may be subjected to higher pollution loads than adjacent lower located regions (Bytnerowicz et al. 2003; Zsigmond and Urák 2011). Mountain slopes of different orientation may differ in both dominating wind direction and intensity, and hence possibly suffer from pollution of different origin and type.

Lichens are slow-growing associations of fungi and green algae or cyanobacteria (Poličnik et al. 2004), known to accumulate some airborne pollutants by both wet and dry deposition (Nash 1996). Lichens differ in their susceptibility to air pollution. For example, they are extremely sensitive to sulphur dioxide causing the most visible consequences referred to as *lichen deserts*. These occur in cities and industrial regions throughout much of the world (Lisowska 2011). Nonetheless, lichens are relatively resistant to metal pollution and their ability to accumulate them is the basis of their common use as bioindicators (Sawicka-Kapusta et al. 2010; Jeran et al. 2007; Kłos et al. 2011; Balabanova et al. 2012). Accumulated trace metals do not interfere noticeably with lichens’ cellular processes, so these organisms are widely used in mapping spatial and temporal patterns of trace metal fallout (Spiro et al. 2004).

## Materials and Methods

The thalli of *Hypogymnia physodes* were collected in July 2012 at slopes of five massifs spread parallel over the whole range of the Outer Beskidy range: Romanka, Pilsko, Polica, Kudłoń and Radziejowa. The geographic position and elevation of each sampling site was determined with a GPS device (Garmin eTrex20) (Table 1).

**Table 1:** Geographical information about studied sites

Site	Mountain range	Mountain height (meters above sea level)	Geographic position of central point of lichens collection area
Romanka	Beskid Żywiecki	1,366	49°34′10′′N; 19°15′28′′E
Pilsko	Beskid Żywiecki	1,557	49°32′37′′N; 19°18′56′′E
Polica	Beskid Żywiecki	1,369	49°37′39′′N; 19°37′19′′E
Kudłoń	Gorce	1,274	49°34′30′′N; 20°11′20′′E
Radziejowa	Beskid Sądecki	1,262	49°27′43′′N; 20°34′44′′E

On every slope 10 samples were collected along ca. 100 m long horizontal transects. Each sample was composed of lichens collected from bark of 2-3 neighboring spruce trees (*Picea abies* L.) using a knife and mixed in a plastic bag.

In the laboratory, the lichens thalli were carefully purged of bark residues, crumbled and dried at 55°C for 4 day to achieve constant mass. Total concentrations of five trace metals (Cd, Cu, Ni, Pb, Zn) were measured after wet digestion of 0.5 g of each sample in 10 mL of a mixture of suprapure concentrated HNO_3_ and HClO_4_ (7:1 v/v) (Sigma-Aldrich, Saint Louis, USA). The mineralization was performed for a week starting form 50°C up to 250°C on a hot plate. To control for efficiency of the digestion, purity of the chemicals and glassware and precision of the analytical equipment, 5 blank samples and 5 replicates of standard certified reference material were processed along with the lichen samples (Trace elements in lichen, BCR^®^—482; IRMM, Geel, Belgium). More information about the used reference material can be found in Kłos et al. (2011). Metal concentrations were measured by atomic absorption spectrometry. Zinc concentrations were measured with flame nebulization (Perkin-Elmer AAnalyst 200, Waltham, MA, USA) and others with graphite furnace (Perkin-Elmer AAnalyst 800, Waltham, MA, USA), because of different ranges of element concentrations. Both instruments used Lumina™ Lamps as emission sources (Perkin-Elmer). Super-clean deionized water was used to set a zero point concentration (Water Pro Ps, AGA Analytical, Warsaw, Poland). Element concentrations were determined with the linear range of standard calibration curves. Quality control checks for elements characteristic mass were performed for every ten analyzed samples. Measured concentrations in mineralized sample were calculated on a dry mass basis, taking into account dilution level. Metal recoveries were determined for spiked samples on the basis of certified values for the respective elements.

The normality criterion for data distribution within groups was checked with a Shapiro–Wilk test and when necessary the data were transformed. The mean values of metal concentrations in lichens from the studied sites were compared with a one-way ANOVA followed by the Tukey-HSD test (*p* < 0.05). In cases where the data were not normally distributed, medians were compared by the Kruskal–Wallis test. Correlation analysis was used to test relationships between concentrations of studied metals, and Pearson’s correlation coefficients were considered significant at *p* < 0.05. Clustering method was used to detect similarity in metal concentrations in lichens from five tested sites (mountains). To standardize metal concentrations, each single outcome for particular metal was divided by a mean concentration of this metal. Clustering method based on nearest neighbor with Euclidean distance. All statistical analyses were performed using the Statgraphics Centurion (StatPoint Technologies Inc., Warrenton VA, USA).

## Results and Discussion

The metal concentrations measured in samples from two massifs, that is Kudłoń and Radziejowa were generally lower than in samples from the other three sites (Table 2). The detection limits for analytical methods used were 0.02, 0.04, 0.51, 0.36 and 8.75 mg kg^−1^ for Cd, Cu, Ni, Pb and Zn, respectively. Metal recoveries were 85, 106 and 95 % for Cu, Pb and Zn, respectively. Differences in mean metal concentrations between sites were found for each element despite the relatively high variance in the data (Table 2).

**Table 2:** Trace metal concentrations in lichen thalli collected on studied sites; mean values (±standard deviation, n = 10)

Site	Metal concentration in lichens (mg kg^−1^)				
	Cd***	Cu***	Ni**	Pb**	Zn*
Romanka	3.6^bc^ (±1.9)	6.1^a^ (±1.3)	0.9^a^ (±0.2)	34.9^a^ (±19.2)	166.9^ab^ (±32.0)
Pilsko	3.7^c^ (±0.6)	9.0^b^ (±1.7)	1.2^ab^ (±0.2)	72.0^b^ (±6.7)	173.6^ab^ (±18.9)
Polica	4.2^c^ (±2.4)	8.7^b^ (±1.8)	1.5^b^ (±0.4)	96.2^b^ (±95.5)	227.3^b^ (±94.8)
Kudłoń	2.1^ab^ (±1.1)	5.7^a^ (±0.9)	1.1^ab^ (±0.3)	34.1^a^ (±4.2)	175.9^ab^ (±26.3)
Radziejowa	1.6^a^ (±0.6)	6.1^a^ (±1.5)	1.0^a^ (±0.7)	28.1^a^ (±12.9)	152.2^a^ (±73.5)

Asterisks indicate significant site differences in mean metal concentration (* *p* < 0.05, ** *p* < 0.01, *** *p* < 0.001)

Differences between stands (columns) are indicated with different letters in superscript (a, b, c)

**Fig. 1:**
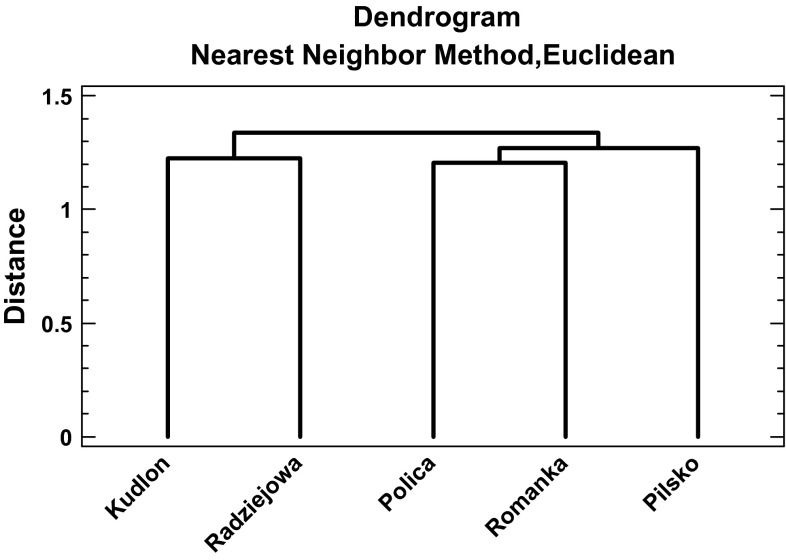
Dendrogram presenting similarity between five massifs in Beskidy Mountains based on metal concentrations (Cd, Cu, Ni, Pb, Zn) in lichens

The five studied sites can be divided into two groups—the more contaminated sites (Pilsko, Polica, Romanka) located on the west of transect and less contaminated located on the east (Kudłoń, Radziejowa) (Fig. 1).

Such results confirm the general pattern of pollution in Poland, with southern and western regions being more polluted than eastern and northern regions. This is a direct repercussion of a combined effect of localization of main industrial centers in Poland and the Czech Republic, and prevalent wind directions at mid-latitudes (Grodzińska et al. 1990).

Any comparisons of concentrations of metals in lichens with data on air pollution available from the EMEP stations for background air pollution is difficult. The EMEP stations measure instantaneous metal concentration in the air, whereas assessing air pollution with lichens reflects long term impingement of air quality on living organisms. It has been proven that heavy metal concentrations in lichen thalli reflect their atmospheric concentrations (van Dobben et al. 2001; Balabanova et al. 2012). However a direct conversion of metal concentrations into actual atmospheric loads is not straightforward. There are multiple reasons for this because accumulation of elements from the atmosphere by lichens is performed via a variety of mechanisms including trapping, ion exchange, extracellular electrolyte sorption, hydrolysis, and intracellular uptake (Conti and Cecchetti 2001). Even if the above issues were minimized and only a single species of lichen was collected, an important source of variability still remains when lichens are collected in situ, namely the age of the thalli. The older thalli are exposed longer and therefore usually contain more pollutants than younger ones (Szczepaniak and Biziuk 2003). However, collection of numerous mixed samples, as done in this study, minimizes this problem. Lichen thalli age is also a source of additional variation in data collected using the transplantation technique, when lichens collected at unpolluted sites are transported and exposed at study sites for a known period of time (Mikhailova 2002). The period of exposure of lichen transplants may influence the vitality of thalli and, consequently, the active process of element uptake (Garty 2001). The success of lichen transplant in monitoring air pollution depends on the level and variability of the background concentration of the elements in the exposed sample (Frati et al. 2005).

The data on metal concentrations in *H. physodes* gathered in this study were compared with data collected in the same laboratory in the framework of long-term environmental monitoring conducted in seven lowland base stations of the integrated nature monitoring system located in regions of different pollution levels in Poland (Sawicka-Kapusta et al. 2010). The mean Cd concentration in lichens collected in our study at five mountain sites in the Beskidy Mountains ranged from 1.6 to 4.2 mg kg^−1^ dwt (Table 2) whereas Cd concentrations in *H. physodes* thalli collected by the base stations were much lower and ranged from 0.4 to 0.9 mg kg^−1^ dwt (Sawicka-Kapusta et al. 2010). The mean Pb concentrations in lichens collected in the Beskidy Mountains ranged from 28.1 to 96.2 mg kg^−1^ dwt (Table 2), whereas in lichens from the base stations varied between 5.1 and 19.9 mg kg^−1^ dwt (Sawicka-Kapusta et al. 2010). The mean Zn concentration in lichens from this study ranged from 152.2 to 227.3 mg kg^−1^ dwt (Table 2) whereas lichens from the base stations ranged from 56 to 121 mg kg^−1^ dwt (Sawicka-Kapusta et al. 2010). Such a comparison suggests much higher air pollution with Cd, Pb and Zn in mountain areas than in lowlands. Even the least polluted mountain sites were characterized by higher metal concentrations than the most polluted lowland site. The concentrations of the remaining two elements, Cu and Ni, were similar in these two studies, and their concentrations in lichens were overlapping.

Metal concentrations in the lichen *Pseudevernia furfuracea* collected in Hăşmaş Mountains (the Eastern Carpathians, Romania) showed air pollution that is lower by an order of magnitude compared to Poland (Zsigmond and Urák 2011). Such a comparison between species needs to be taken with caution, because lichen species differ in their abilities to trap metals. The metal concentrations measured by Zsigmond and Urák (2011) were up to 0.2 mg kg^−1^ dwt for Cd, 13.7 mg kg^−1^ dwt for Pb and 84.4 mg kg^−1^ dwt for Zn. Only Cu levels were similar to those measured in Poland. Even though the Eastern Carpathians are one of the least contaminated regions in Europe, the authors still considered the studied region as slightly polluted (Zsigmond and Urák 2011).

Because of strong correlations between metal concentrations in lichen samples, we deduced that all five analyzed trace metals had similar sources (Table 3), most likely industry. The highest correlation was observed for lead and zinc, which are known to occur jointly in ore deposits and are processed in smelters in south-west Poland and in the Ostrava region in the Czech Republic, approximately 150 km away from the western end of the study transect. These areas are known to be heavily polluted with metals to an extent affecting biodiversity and ecosystem functions (Klimek and Niklińska 2007; Azarbad et al. 2013).

**Table 3:** Correlations between metal content in individual lichen samples

	Cu	Ni	Pb	Zn
Cd	0.2880*	0.3226*	0.4362**	0.5306***
Cu		0.3509*	0.5789***	0.3841**
Ni			0.4395**	0.3125*
Pb				0.8278***

Pearson’s correlation coefficients (range from −1 to +1) are followed in parentheses by asterisks showing statistical significance of the estimated correlations (* *p* < 0.05, ** *p* < 0.01, *** *p* < 0.001)

The strongest correlations are presented in bold

Also, Poličnik et al. (2004) found increased concentrations of Pb and Zn in the lichen *H. physodes* in the vicinity of Zn and Pb smelters in Slovenia (Žerjav site). They calculated enrichment rates as high as 100 mg per month for Pb and 12 mg per month for Zn (Poličnik et al. 2004). Similarly, a Cu smelter was also the major source of Cu in *H. physodes* in Macedonia (Balabanova et al. 2012). They found mean Cu concentration in lichens as high as 12 mg kg^−1^ with maximum value of 130 mg kg^−1^, which were much higher than the corresponding values for Cu in our study (Table 2).

In summary, we showed that the Beskidy Mountains are moderately polluted with Cd, Pb and Zn. Concentrations of these elements were higher than in the lowlands, whereas Ni and Cu were comparable. Elevated concentrations of some metals in lichens may result from more massive atmosphere movements and wet deposition in mountain areas compared to lowlands.

Our study fills a gap in the national monitoring program and shows the existence of heavy metal pollution in regions regarded as unpolluted. Lichens are especially useful for long-term evaluation of air pollution, giving a good overview about the condition of the environment.
